# Preconditioning with L-Ala-Gln reduces the expression of inflammatory markers (TNF-α, NF-κB, IL-6 and HO-1) in an injury animal model of cerebrovascular ischemia in *Meriones unguiculatus* (gerbils)^1^

**DOI:** 10.1590/s0102-865020200060000001

**Published:** 2020-07-20

**Authors:** Leidelamar Rosário Alves de Oliveira, Andréa de Oliveira Albuquerque, Cícero Igor Simões Moura Silva, Jussara Mathiele Silva, Meyssa Quezado de Figueiredo Cavalcante Casadevall, Orleancio Gomes Ripardo de Azevedo, Vilma Leite de Souza Pires Albuquerque, Paulo Roberto Leitão de Vasconcelos

**Affiliations:** I Master, Department of Medical Surgical Science, Universidade Federal do Ceará (UFC), Fortaleza-CE, Brazil. Statistical analysis, analysis and interpretation of data, technical procedures.; II PhD, Medical Surgical Science, UFC, Fortaleza-CE, Brazil. Statistical analysis, analysis and interpretation of data, technical procedures.; III Fellow Master degree, Postgraduate Program in Pharmacology, Department of Physiology and Pharmacology, UFC, Fortaleza-CE, Brazil. Manuscript preparation.; IV PhD, Department of Medical Surgical Science, UFC, Fortaleza-CE, Brazil. Manuscript preparation and writing, critical revision.; V Associate Professor, Centro Universitário Christus (UNICHRISTUS), Fortaleza-CE, Brazil. Scientific and intellectual content of the study.; VI PhD, Department of Medical Surgical Science, UFC, Fortaleza-CE, Brazil. Critical revision, final approval.

**Keywords:** Anti-Inflammatory Agents, Glutamine, Immunohistochemistry, Dietary Supplements

## Abstract

**Purpose:**

To evaluate the neuroprotective effect of L-alanyl-glutamine in a gerbil model of brain ischemia-reperfusion injury based on immunohistochemical quantification of pro-inflammatory and cell activation biomarkers (TNF-α, NF-κB, IL-6 and HO-1).

**Methods:**

Male gerbils weighing 100-180 g were pretreated with either 0.75 g/kg L-Ala-Gln (n=18) or 2.0 mL saline (n=18) administered i.v. 30 minutes before the bilateral ligation of the common carotid artery during 15 min and then the ligation was removed. Under anesthesia with urethane, brain tissue was harvested at 0 min (T_0_), 30 min (T_30_) and 60 min (T_60_) after reperfusion. The tissue was embedded in 10% formalin overnight and 4-μm sections were prepared for immunostaining with monoclonal antibodies. Immunostained cells were counted by optical microscopy. The statistical analysis used mean values based on 4 sections.

**Results:**

The pretreatment with L-Ala-Gln animal group 1 demonstrated significantly lower levels of TNF-α, NF-κB and IL-6. On the other hand, the levels of HO-1 were significantly higher, suggesting a protective role in model of brain ischemia-reperfusion injury.

**Conclusion:**

These findings suggest a protective effect of L-Ala-Gln by decreasing levels of TNF-alpha, IL-6 and NF-κB and Increasing levels of HO-1.

## Introduction

Cerebrovascular diseases are currently the third-most important cause of death in several developed and developing countries. The European incidence of cerebral infarction is 1.35-2.2 / 1,000 inhabitants, and 83% of cases are associated to ischemic etiology^1^. According to the National Institute of Health, cerebrovascular diseases affect about 500,000 people every year in the U.S., with a mortality rate of 20-30% and a similar rate of severe disability^2^.

Cerebral ischemia is a reduction or total absence of oxygen and other metabolic substrates in the brain due to total or partial obstruction of the blood supply^3^. Reperfusion after brain ischemia increases the levels of pro-oxidant reactive oxygen species (ROS) in the brain tissue and may lead to neuronal injury as ROS interact directly with macromolecules (including proteins, lipids and DNA) or indirectly affect cellular signaling pathways and the regulation of gene expression^4^.

The triggering and maintaining of the inflammatory state depend on several known mediators secreted by activated cells at the site of injury^5,6^. IL-6 is a cytokine and its physiological role in injury-induced inflammation shows a potent pro-inflammatory role^7^.

TNF-α acts on endothelial cells by promoting the migration of neutrophils and plays a key role stimulating endothelial cells to produce and release chemokines^8^.

The nuclear factor *kappa* B (NF-κB) regulates the expression of genes essential for inflammation, cell survival, proliferation and apoptosis^9^. Microsomal heme oxygenase-1 (HO-1) is activated by oxidative stress or by the presence of proinflammatory cytokines, endotoxins, heme and nitric oxide^10,11^.

Several reports have been published investigating cerebral ischemia using animal models. Several species have been tested^11^; among these, the Mongolian gerbil (*Meriones unguiculatus*) is an adjusted methodological option due to its high susceptibility to experimental cerebral ischemia induced by ligation of the common carotid artery^12^.

Numerous studies have shown that L-alanyl-L-glutamine is an essential amino acid, which is actively transported and metabolized in all animal tissues^13^; the glutamate-glutamine cycle between glial cells and nerve endings is maintained through the uptake of gamma-amino butyric acid and glutamate by astrocytes. These amino acids are converted to glutamine^14^. Disturbances in synthesis may result in the accumulation of glutamate in glial cells leading to neurotoxicity.

Studies by Kanoria^12^ have shown that preconditioning with L-Ala-Gln protects against ischemia-reperfusion injury in several organs. Thus, the aim of this study was to evaluate the role of L-Ala-Gln to protect against brain ischemia-reperfusion injury in a gerbil model evaluated through immunohistochemistry (IHC) for the IL-6, TNF-α, NF-κB and HO-1.

## Methods

The study was conducted in accordance with the International Guiding Principles for Biomedical Research Involving Animals (1990). The study protocol was approved in 2008 by the Research Ethics Committee (CEPA) of Universidade Federal do Ceará (UFC), #127/07. The gerbils were supplied by the experimental animal facility at Centro Universitário Christus and used at the laboratory of experimental surgery (UFC School of Medicine).

### *Experimental design*

The sample consisted of 36 healthy, well-nourished male gerbils (*Meriones ungiculatus*) aged 8-16 months and weighing 100-180 g. The sample was divided into two groups: in Group 1 (n=18), the animals were pretreated with saline (control) and submitted to ischemia and reperfusion. In Group 2 (n=18), the animals were pretreated with L-Ala-Gln and submitted to ischemia and reperfusion. Each group was divided into three subgroups (n≥4) according to the time following reperfusion: 0 min (T_0_), 30 min (T_30_) and 60 min (T_60_)^15^.

The animals were anesthetized with an intraperitoneal injection of urethane (1.5 g/kg) and submitted to osteotomy with a double-sided flexible grindstone attached to an electric motor, followed by the harvesting of brain tissue^15,16^. Removal of internal pyramidal layer of the parietal region was performed using a method by Pires and cols. At the end of the procedure, the animals were euthanized by cervical dislocation.

### *Brain ischemia protocol*

The surgical procedure started with trichotomy followed by an incision in the ventral region of the neck; the muscular and subcutaneous tissues were dissect with individualization and bilateral isolation of the common carotid arteries (CCAs) at 0.5 cm of bifurcation in the external carotid arteries and internal (occlusion point), followed by identification and clamping of the arteries, with bulldog type vascular clips, for a period of 15 minutes of ischemia followed by two reperfusion periods T30 and T60.

### *Chemicals*

L-Ala-Gln was purchased from Fresenius Brazil. The 0.9% saline solution was purchased from Gaspar Viana and administered in a standardized volume of 2.0 mL.

### *IHC analysis*

The brain tissue was submitted to immunohistochemical analysis with streptavidin-biotin to quantify TNF-α, NF-κB, IL-6 and HO-1. Initially, the tissue was fixed in 10% formalin for 24 hours, followed by embedding in paraffin. Sections measuring 4 μm were prepared with a microtome and placed on slides with poly-L-lysine, then dehydrated and hydrated with decreasing concentrations of alcohol and xylene. After heat-induced antigen retrieval (10min in citrate buffer pH=6.0), blocking with 3% hydrogen peroxide and rinsing with phosphate buffer solution (PBS), the samples were incubated overnight at 4ºC, according to manufacturer´s. Primary antibodies against IL-6 and TNF-α (goat polyclonal). NF-kB and HO-1 (mouse monoclonal) were used (1:200 diluted in 1 x PBS containing 5% BSA) (Santa Cruz, Texas, USA). The negative controls were treated with BSA but not with primary antibody.

After overnight incubation, the slides were washed in PBS and incubated with secondary mouse-anti-goat IgG-HRP for TNF-alpha and IL-6; and goat-anti-mouse for NF-kB and HO-1 both (1:100 diluted in 1 x PBS containing 5% BSA) (Santa Cruz, Texas, USA). Then, the slides were washed in PBS, and streptavidin- ABC complex conjugate (Santa Cruz) was added, followed by drying and the addition of 3,3-diaminobenzidine (Dako). Finally, the slides were mounted using Entellan (São Paulo, Brazil) and coverslips.

The evaluation of slides was performed by counting immunostained nerve cells, both neurons and neuroglia cells, observed at 400X magnification in an Olympus BX41 optical microscope. Ten fields of each slice were observed (x40, 10 oculars, 0.5024mm^2^ per field), with a total of 4 cuts per group, always trying to start with the internal pyramid layer. The analyses were performed by a pathologist. After the examiner’s analysis, the immunohistochemistry findings were sent for statistical tests of concordance.

### *Statistical analysis*

The statistical analysis was performed with the software SPSS (v.17.0). Quantitative variables were initially analyzed with the Shapiro-Wilk test to verify the normality of the distribution. Having confirmed normality in all cases, mean values and standard deviations were calculated based on four sections. The groups and reperfusion times were compared with ANOVA and the post-hoc Tukey test. The level of statistical significance was set at 5% (*p*<0.05).

## Results

### *Immunohistochemistry for TNF-α*

**Figure 1:**
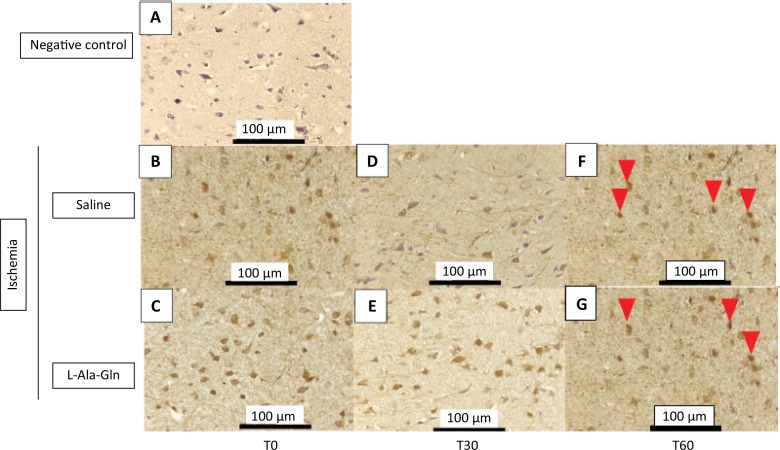
Mean TNF-α levels (± SEM) in gerbil brain tissue according to time (T0, T30 and T60) and pretreatment with saline 0.9% *vs*. L-Ala-Gln (0.75g/kg). Representative photographs of, effect of pretreatment with L-Ala-Gln on internal pyramidal layer of parietal area, on immunoreactivity of TNF-alpha in the pyramidal layer in parietal cortex. (A) negative control (B, D and F) transient cerebral ischemia and saline 0,9% NaCl; C, E and G transient cerebral ischemia and L-Ala-Gln (0.75 g/kg): The figures B and C; D and E; and F and G are from T0, T30 and T60 minutes after the brain ischemia. Arrowheads show immunostained cells. Each group contained at least 4 animals. Bar=100 µm.

The IHC TNF-alpha immunostained cells in the internal pyramidal layer of parietal region of animals that received L-Ala-Gln were significantly reduced after 60 minutes when compared to control group (saline 0.9% NaCl) (Fig. 1). TNF-alpha levels were significantly decreased after 60 minutes of ischemia (p=0.001) (Table 1). The immunoreactivity after 30 minutes showed a discrete reduction (p>0.05) on immunostained cells in L-Ala-Gln group in comparison to control group.

**Table 1:** Immunoreactivity for TNF-a in brain tissue sections from internal pyramidal layer of the cerebral cortex positive cells for each field on 0, 30 and 60 minutes.

Time (min)	Ischemia + Saline	Ischemia + L-Ala-Gln	p
	Mean ± SEM	Mean ± SEM	
0	30.3±36.0	3.5±7.0	p=1.000
30	333.0±395.6	3.5±7.0	p=0.503
60	1015.5±102.8	3.5±7.0	p=0.001*

The number of TNF-alpha immunoreactive neurons in saline and L-Ala-Gln groups. The morphological analysis was performed evaluating the immunostained central nervous system nerve cells (neurons and neuroglia cells), observed at 400X magnification in an Olympus BX41 optical microscope. Ten fields of each cut were observed, with a total of 4 cuts per group, always trying to start with the internal pyramid layer (p<0.05).

### *Immunohistochemistry for NF-kB*

**Figure 2:**
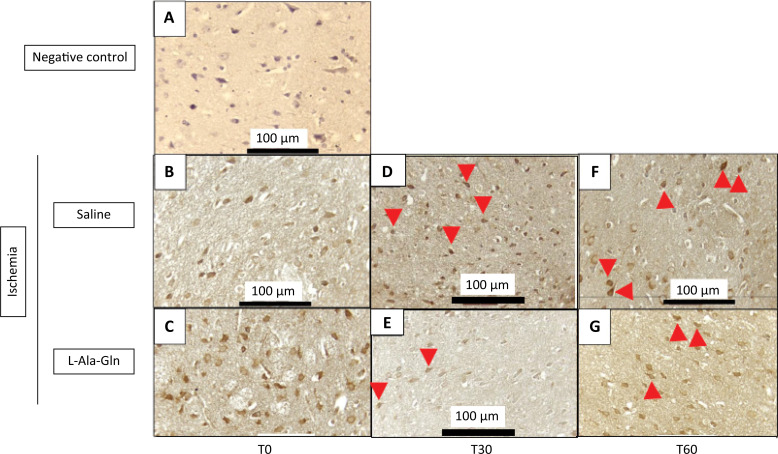
Mean NF-κB levels (± SEM) in gerbil brain tissue according to time (T0, T30 and T60) and pretreatment with saline 0.9% *vs*. L-Ala-Gln (0.75g/kg). Representative photographs of, effect of pretreatment with L-Ala-Gln on internal pyramidal layer of parietal area, on immunoreactivity of NF-kB in the pyramidal layer in parietal cortex. (A) negative control (B, D and F) transient cerebral ischemia and saline 0,9% NaCl; C, E and G transient cerebral ischemia and L-Ala-Gln (0.75 g/kg): The figures B and C; D and E; and F and G are from T0, T30 and T60 minutes after the brain ischemia. Arrowheads show immunostained cells. Each group contained at least 4 animals. Bar=100 µm.

The animals that received L-Ala-Gln demonstrated significantly reduced positive-IHC neurons of NF-κB levels on the internal pyramidal layer of the parietal region, detected through immunohistochemistry in the cortex of parietal region, after 30 and 60 minutes, respectively when compared to saline (0.9% NaCl) group (p=0.001) (Table 2). In addition, the NF-kB immunoreactivity was weaker in L-Ala-Gln group compared to saline group levels (Fig. 2).

**Table 2:** Immunoreactivity for NF-kB in brain tissue sections from internal pyramidal layer of the cerebral cortex positive cells for each field on 0, 30 and 60 minutes.

Time (min)	Ischemia + Saline (0.9%)	Ischemia + L-Ala-Gln (0,75g/kg)	p
**Mean ± SEM**	**Mean ± SEM**
0	463.0±535.0	450.3±523.8	p=1.000
30	1234.8±144.9	247.5±495.0	p=0.0001*
60	1286.3±85	217.8±435.5	p=0.0001*

The number of NF-kB immunoreactive neurons in saline and L-Ala-Gln groups. The morphological analysis was performed evaluating the immunostained central nervous system nerve cells (neurons and neuroglia cells), observed at x400 magnification in an Olympus BX41 optical microscope. Ten fields of each cut were observed, with a total of 4 cuts per group, always trying to start with the internal pyramid layer. L-Ala-Gln *vs*. Saline group p<0.0001 at T30 and T60 after ischemia.

### *Immunohistochemistry for IL-6*

**Figure 3:**
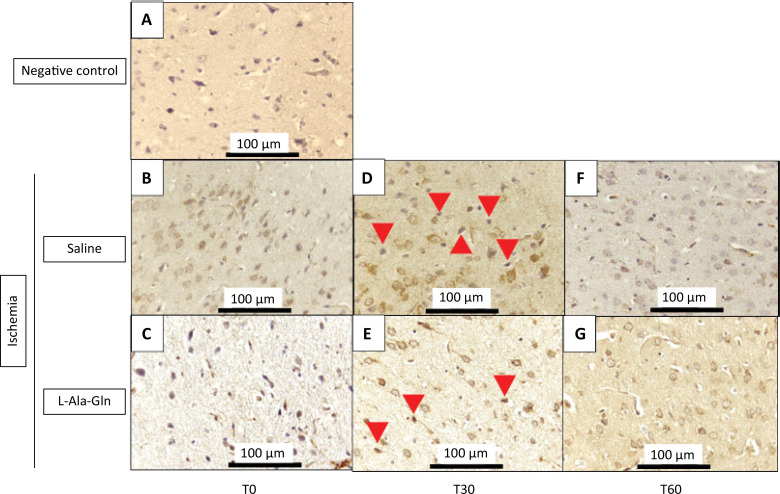
Mean IL-6 levels (± SEM) in gerbil brain tissue according to time (T0, T30 and T60) and pretreatment with saline 0.9% *vs*. L-Ala-Gln (0.75g/kg). Representative photographs of, effect of pretreatment with L-Ala-Gln on internal pyramidal layer of parietal area, on immunoreactivity of IL-6 in the pyramidal layer in parietal cortex. (A) negative control (B, D and F) transient cerebral ischemia and saline 0,9% NaCl; C, E and G transient cerebral ischemia and L-Ala-Gln (0.75 g/kg): The figures B and C; D and E; and F and G are from T0, T30 and T60 minutes after the brain ischemia. Arrowheads show immunostained cells. Each group contained at least 4 animals. Bar=100 µm.

The IL-6 immunostained positive cells had demonstrated a significant decrease after 30 minutes of ischemia injury on internal pyramidal layer of the parietal area from animals treated with L-Ala-Gln (Fig. 3). Although, after 60 minutes, the means of both groups are quite different (316.0 and 28.5); however, it cannot demonstrate the significance due to the standard error of mean (SEM) of the saline group (p=0.033) (Table 3).

**Table 3:** Immunoreactivity for IL-6 in brain tissue sections from internal pyramidal layer of the cerebral cortex positive cells for each field on 0, 30 and 60 minutes.

Time (min)	Ischemia + Saline (0.9%)	Ischemia + L-Ala-Gln	p
	Mean ± SEM	Mean ± SEM	
0	311.0±246.5	14.8±29.5	p=0.817
30	536.3±119.8	9.0±18.0	p=0.033*
60	316.0±365.4	28.5±57.0	p=0.591

The number of IL-6 immunoreactive neurons in saline and L-Ala-Gln groups. The morphological analysis was performed evaluating the immunostained central nervous system nerve cells (neurons and neuroglia cells), observed at x400 magnification in an Olympus BX41 optical microscope. Ten fields of each cut were observed, with a total of 4 cuts per group, always trying to start with the internal pyramid layer. L-Ala-Gln *vs*. Saline group p<0.033 at T30 after ischemia.

### *Immunohistochemistry for HO-1*

**Figure 4:**
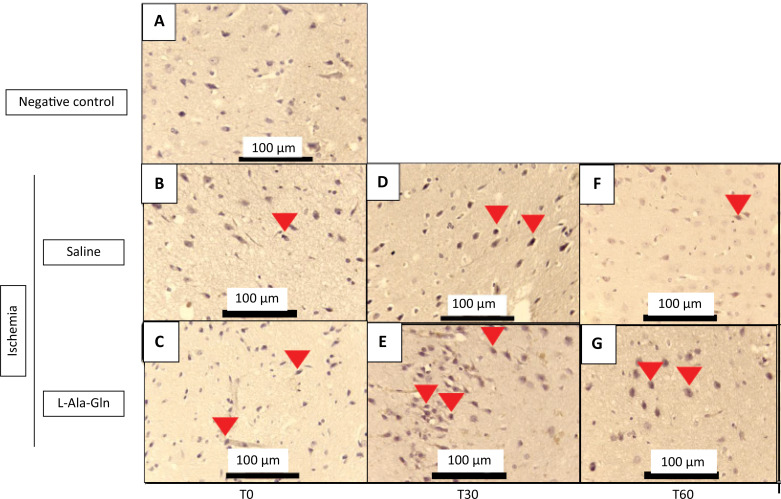
Mean HO-1 levels (± SEM) in gerbil brain tissue according to time (T0, T30 and T60) and pretreatment with saline 0.9% *vs*. L-Ala-Gln (0.75g/kg). Representative photographs of, effect of pretreatment with L-Ala-Gln on internal pyramidal layer of parietal area, on immunoreactivity of HO-1 in the pyramidal layer in parietal cortex. (A) negative control (B, D and F) transient cerebral ischemia and saline 0,9% NaCl; C, E and G transient cerebral ischemia and L-Ala-Gln (0.75 g/kg): The figures B and C; D and E; and F and G are from T0, T30 and T60 minutes after the brain ischemia. Arrowheads show immunostained cells. Each group contained at least 4 animals. Bar=100 µm.

The HO-1 marker had demonstrated an significant increase on immunostained positive cells of internal pyramidal layer of parietal area from tissue brain of gerbils treated with L-Ala-Gln, after 0, 30 and 60 minutes of ischemia and reperfusion injuries in animals treated with L-Ala-Gln (p=0.015), (p=0.013) and (p=0.007) respectively, in comparison to gerbils that were administered saline 0.9% NaCl, observed in internal pyramidal layer of parietal region (Fig. 4, Table 4).

**Table 4:** Immunoreactivity for HO-1 in brain tissue sections from internal pyramidal layer of the cerebral cortex positive cells for each field on 0. 30 and 60 minutes.

Time (min)	Ischemia + Saline (0.9%)	Ischemia + L-Ala-Gln (0.75g/kg)	p
	Mean ± SEM	Mean ± SEM	
0	3.4±9.7	9.8±13.0	p=0.015*
30	6.8±13.0	11.3±13.7	p=0.013*
60	7.5±13.7	18.1±13.5	p=0.007*

The number of HO-1 immunoreactive cells in saline and L-Ala-Gln groups. The morphological analysis was performed evaluating the immunostained central nervous system cells (neurons and neuroglia cells), observed at 400X magnification in an Olympus BX41 optical microscope. Ten fields of each cut were observed. with a total of 4 cuts per group. always trying to start with the internal pyramid layer. The p=0.015, p=0.013, and p=0.007 *vs* 0.9% Na Cl control group after 0, 30 and 60 minutes of reperfusion respectively.

## Discussion

In this study, we found immunohistochemical evidence of the protective effect of L-Ala-Gln on the internal pyramidal layer of the parietal area of gerbil brain tissue exposed to ischemia and reperfusion injuries based on the quantification of inflammation and cell activation biomarkers (TNF-α, NF-κB, IL-6 and HO-1). In 2011, Pires and cols. using the same bilateral occlusion protocol of cerebral ischemia/reperfusion demonstrated that precondition with L-Ala-Gln reduced the oxidative stress in cerebral tissue^15^. Thus, this study evaluates the inflammatory aspect of cerebral ischemia/reperfusion.

The finding of significantly lower TNF-α levels in L-Ala-Gln group at T_60_ suggests L-Ala-Gln has a neuroprotective effect preventing cellular damage, on internal pyramidal layer of parietal area, induced by cytokines and proinflammatory mediators released in association with inflammatory microvascular injury and ROS-mediated cytotoxicity^17^.

A redox-sensitive transcription factor, NF-κB activates the inflammatory transcription cascade, regulating an array of inflammatory genes in addition to certain mediators with anti-inflammatory action. NF-κB has therefore been proposed as a target for cell protection against oxidative stress, pro-inflammatory factors and sclerosis in several sites, including the myocardium and the brain^18^. Our finding of significantly reduced levels of NF-κB in preconditioned animals with L-Ala-Gln at T_60_ is in concordance of other reports investigating the neuroprotective action of L-Ala-Gln and its interaction with glutaminergic NF-κB-dependent pathways^18^.

IL-6 is a multifunctional cytokine produced by several cell types, especially cells of the mononuclear phagocyte system. It plays an important role in lymphocyte (CD4+) differentiation, immunoglobulin secretion, formation of multipotent cell colonies in the bone marrow, and several proteins involved in the acute phase of systemic inflammation^19^.

The significantly lower IL-6 levels observed in L-Ala-Gln group at T_30_ suggests its protective against brain cell damage induced by cytokines and proinflammatory mediators released in association with microvascular injury. These findings agree with those of other studies employing cerebral ischemia/reperfusion models^19,20^.

Some studies have shown that the induction of HO-1 promotes cellular protection against oxidative injury through different mechanisms, such as by controlling intracellular levels of free heme (an anti-oxidant), producing biliverdin (an anti-oxidant), improving perfusion of nutrients via the release of CO, and inducing ferritin synthesis through the release of free iron^21,22^.

Furthermore, low heme concentrations may have anti-inflammatory and cytoprotective effects by increasing the HO-1 expression and stimulating the formation of HO-1 and its products, such as CO and biliverdin. In the present study, HO-1 levels at T_0_, T_30_ and T_60_ were significantly higher in tissues from animals preconditioned with L-Ala-Gln, as shown elsewhere in the literature^21,23-27^.

L-Aln-Gln has been investigated extensively in order to evaluate tissue injury pathways and mechanisms in target organs in ischemia-reperfusion models^13^. The studies published so far provide strong evidence of a cytoprotective effect of L-Ala-Gln in various cell types and of the molecular and biochemical-signaling pathways implicated in antioxidant defense and the (probably sclerotic) anti-inflammatory action of L-Aln-Gln.

Preconditioning with L-Aln-Gln has shown to reduce the extension of myocardial cell damage associated with ischemia-reperfusion injury in experimental models. L-Aln-Gln is believed to inhibit the harmful effects of neuronal NO synthetase by nitrergic route and thereby inhibit glutamine synthetase. The blocking of NO synthesis may involve other enzymes such as glutamine synthase^28^. It should be pointed out that the main metabolic changes are observed during the first minutes of reperfusion, indicating they represent a reaction to early reperfusion-induced oxidative stress^16^.

The epidemiological data of the non-transmissible diseases such as cerebrovascular ischemia increased in the last decade, requiring the development of a new tool to prevent or to reduce the potential negative effects of this kind of injury; thus, a translational approaching for this study could be done. Probably, the adoption of a diet rich in amino acids like Alanine and Glutamine could be a protective factor reducing the worst damages on neuronal tissue that led the patients to a several disabilities in many cases.

Further studies including a larger array of inflammatory and cell activation biomarkers are required to confirm the efficacy and safety of preconditioning with L-Ala-Gln against the deleterious effects of brain ischemia and reperfusion.

## Conclusion

Preconditioning with L-Ala-Gln has a potentially protective role against inflammation induced by brain ischemia and reperfusion.
